# Daily interactions with care recipients and cardiovascular reactivity among dementia caregivers: The buffering role of friend interactions

**DOI:** 10.1002/alz.70281

**Published:** 2025-05-15

**Authors:** Yee To Ng, Anna Kratz, Angela Turkelson, Kira Birditt

**Affiliations:** ^1^ Institute for Social Research University of Michigan Ann Arbor Michigan USA; ^2^ Department of Physical Medicine and Rehabilitation University of Michigan Ann Arbor Michigan USA

**Keywords:** ambulatory cardiovascular reactivity, caregiver‐care recipient interactions, ecological momentary assessment, friendship, heart rate

## Abstract

**INTRODUCTION:**

Caregiver–care recipient (CR) interactions are central to caregiving, yet little is known about dementia caregivers’ daily experiences with CRs. This study examines within‐day effects of time interacting with CRs and interaction quality on caregivers’ heart rate (HR) and whether friend interactions buffer these effects.

**METHODS:**

Dementia caregivers (*N *= 221) completed baseline interviews and 5‐day ecological momentary assessments (EMAs), reporting time and interaction quality with CRs and friend interactions every 3 hours while wearing heart monitors.

**RESULTS:**

Within‐day analyses revealed time with CRs was associated with increased HR, primarily due to negative interactions. Friend interactions buffered the adverse effects of time interacting with CR and negative CR interactions on elevated HR. Moreover, positive CR interactions were linked to lower HR when paired with friend interactions.

**DISCUSSION:**

Findings provide insights into interventions that reduce negative exchanges within the caregiver–CR dyad and friend‐based programs, which could enhance cardiovascular health among dementia caregivers.

**Highlights:**

- More time interacting with the care recipient (CR) was associated with an elevated heart rate (HR).
- Negative daily experiences with CRs are more salient in contributing to caregivers’ cardiovascular strain.
- Friend interactions can temporarily buffer the adverse effects mentioned above.
- Positive CR interactions linked to lower HR when paired with friend interactions.
- Socializing with friends can be beneficial, even if some interactions are negative.

## 1 BACKGROUND

Dementia caregiving is uniquely stressful because of care recipients’ (CR) cognitive decline, behavioral changes, and increased dependency on caregivers.^1^, ^2^ These challenges place dementia caregivers at greater risk for adverse health outcomes than caregivers for other conditions.^3^, ^4^ Cardiovascular health is a particular concern, with cross‐sectional and longitudinal studies linking dementia caregiving to increased cardiovascular risks. These risks include cross‐sectional outcomes such as elevated blood pressure, increased heart rate (HR), and adverse inflammatory/biochemical markers (e.g., increased interleukin‐6, C‐reactive protein [CRP], and salivary cortisol), as well as longitudinal outcomes such as the onset or diagnosis of cardiovascular disease (CVD), including hypertension, stroke, and coronary heart disease.^5^, ^6^

Given the consistent link between caregiving and increased cardiovascular risks, it is critical to identify modifiable factors that can disrupt this link.^7^ The stress buffering model of social relationships^8^ posits that social ties can reduce stress reactivity. Friendships are widely recognized as a buffer against the negative impact of life experiences on health across various populations. Yet, the unique stress‐buffering role of friendships has been relatively unexamined in dementia caregiving contexts, as studies typically combine family and friends.^9^, ^10^, ^11^, ^12^, ^13^ Indeed, Lilly et al. found that one‐third of dementia caregivers in the National Caregivers Training Study mentioned their friends during open‐ended interviews, which were designed to explore their caregiving experiences and support networks.^14^ The majority of these mentions were related to emotional support or social integration for the caregivers, highlighting friendships’ role in reducing stress and enhancing social integration. Similarly, friends, unlike family members, typically do not live in the same household as caregivers and may serve as a link to the “outside world.”^15^ While some friends may provide in‐home respite assistance,^16^ others may not be involved in caregiving but instead offer companionship through shared leisure activities.^17^ In either case, friends support caregivers in taking a break from caregiving, allowing them to recharge and relax. This study focuses on how friendships may alleviate the physiological responses to caregiving stress. Although the direct effects of friend interactions on dementia caregivers’ cardiovascular health are important, the buffering role of friendships is crucial for understanding how they protect caregivers from stress and stress‐related cardiovascular risks. Moreover, this study does not aim to compare the buffering roles or strength of friends, family, and others, but instead has a unique focus on friends, as their interactions may be more flexible and changeable than family interactions, offering distinct opportunities for intervention.

Decades of research have operationalized or explored key aspects of dementia caregiving, such as comparisons between dementia caregivers and non‐dementia caregivers or non‐caregivers, caregiver stress, distress related to CR problem behaviors, and the intensity and duration of caregiving.^6^ Considering the centrality of caregiver–CR interactions in caregiving,^18^ this study pioneers an investigation into how the daily amount of time interacting with CRs and the quality of these interactions influence ambulatory cardiovascular health among dementia caregivers. We examine real‐time social interactions and HR in natural environments using ecological momentary assessments (EMAs) and ambulatory heart monitors. Elevated HR relative to an individual's average is considered an acute stress response,^19^ which is associated with increased risks of hypertension, CVD, and mortality.^20^, ^21^, ^22^ This innovative multi‐method approach allows for nuanced analyses of the temporal links between interactions with CR, friends, and cardiovascular health, addressing the limitations of cross‐sectional surveys or lab‐based studies.

**RESEARCH IN CONTEXT**

1. **Systematic review**: The authors reviewed articles on dementia caregiving, cardiovascular health, and adult friendships using Google Scholar. They referenced studies on increased cardiovascular risks for dementia caregivers and the role of friendships in caregiving in the context of dementia caregiving.
2. **Interpretation**: The findings revealed that spending more time interacting with care recipients (CRs) than usual was associated with elevated heart rate (HR), primarily driven by negative interactions with CRs. However, interactions with friends mitigated the adverse effects of more time interacting with CRs and negative CR interactions on HR. Furthermore, friend interactions combined with positive CR interactions resulted in lower HR, highlighting the important role of friends in alleviating some of the stress linked to caregiving.
3. **Future directions**: We call for targeted interventions to facilitate more friend interactions for dementia caregivers and positive interactions within the dementia caregiver–CR dyad; such interventions could offer stress reduction and enhance cardiovascular health.

Together, this study addresses two key research questions along with corresponding hypotheses:

1. Is the amount of time spent interacting with CRs and their interaction quality linked to elevated HR in daily life among dementia caregivers?

**H1**: Longer time spent interacting with CRs will be linked to elevated HR, especially during negative interactions with CRs.

2. Do friend interactions and their interaction quality buffer the effects of time and quality of CR interactions on caregivers’ HR?

**H2**: Friend interactions, particularly positive ones, will buffer the impact of longer and more negative interactions with CRs on HR.

## 2 METHODS

### 2.1 Sample and procedures

Data were drawn from the Stress and Well‐Being in the Everyday Lives of Caregivers (SWELCare) Study, collected from December 2021 to August 2024. Eligible participants included 221 Black and White adults in the states of Michigan or Ohio who provided unpaid care to an adult family member or friend with dementia (96.4% were 55 years old or older). The inclusion criteria specified that caregivers must be the primary caregiver, reside with the CR, identify as non‐Hispanic Black or non‐Hispanic White, and be able to speak and read English. While CRs were not required to have a formal dementia diagnosis, they needed to have a caregiver‐reported history of worsening memory and difficulty with daily activities. This was assessed using the AD8 Dementia Screening Interview,^23^ a brief questionnaire designed to detect signs of dementia. The AD8 consists of eight yes/no questions evaluating changes in cognitive function over time. To meet eligibility criteria, caregivers had to report that their CR scored at least two items on the AD8. This addressed racial disparities in formal diagnosis rates, as Black Americans are less likely to receive a formal diagnosis.^24^, ^25^, ^26^ For CRs with a formal dementia diagnosis from a doctor, caregivers were asked to specify the type of dementia (e.g., AD, vascular dementia, Lewy body dementia, Parkinson's disease dementia, Huntington's disease, etc.). Eligible caregivers completed a 90‐ to 120‐min baseline interview on the phone, providing information on demographics and caregiving background, followed by a Zoom or phone training session on learning how to complete mobile surveys and use a heart monitor. For 5 days, caregivers wore a heart monitor and completed six brief EMAs on a study‐provided mobile device. Written consent was obtained from all participants. Caregivers were paid up to $340: $50 for the baseline assessment, $50 per day for each day of the 5‐day EMA and ambulatory heart monitoring, and a $40 bonus for completing all aspects of the study. The study protocol was approved by the University of Michigan Ethics Review Board, and all participants provided informed consent.

### 2.2 EMA measures

Participants completed EMA surveys on study‐provided phones at scheduled times (interval‐based measurement): upon waking, and then at 9 am, 12 noon, 3 pm, 6pm, and before bed, over a period of 5 days. Since the waking survey did not include questions about social interactions, these surveys were excluded from the analyses.

**Time spent interacting with the CR**. Every 3 h, caregivers reported the amount of time they were in contact with the CR over the past 3 h, including in‐person interactions, phone calls, texts, emails, social media, or video chats. They chose from 0–3 h and rounded minutes to 0, 15, 30, or 45. A continuous variable for time interacting with CR *in minutes* was derived.

**Levels of positive and negative interaction quality with the CR**. If caregivers reported any time interacting with CR in the past 3 h, they rated the interaction on two questions: the extent to which interacting with CR was positive/enjoyable from 1 (*not at all*) to 5 (*a great deal*) and the extent to which it was irritating/hurtful/annoying/stressful from 1 (*not at all*) to 5 (*a great deal*). The levels of positive and negative interactions with CRs were treated as two separate continuous variables.

**Social interactions with friends**. Other than CR, caregivers reported social interactions occurred in the past 3 h, including in‐person interactions, phone calls, texts, emails, social media, and video chats. We coded whether caregivers had any interactions with friends within each 3‐h interval, coded as 1 (*yes*) or 0 (*no*).^27^, ^28^

**Positive and negative interaction quality with friends**. Of the interactions caregivers had over the past 3 h, they indicated which ones were positive/enjoyable and which ones were irritating/hurtful/annoying/stressful. Two binary variables were created to indicate whether there was any positive interaction with friends 1 (*yes*) 0 *(no*) and any negative interaction with friends 1 (*yes*) 0 (*no*) within each 3‐h interval.^28^ Notably, these two binary variables were not mutually exclusive, as caregivers could report both positive and negative interactions with friends, or neither, within each 3‐h interval.

### 2.3 Cardiovascular measures

Caregivers wore an electrocardiogram (ECG) heart monitor (the BodyGuardian mini by Preventice Inc.; United States Food and Drug Administration [FDA] approved and commercially available) with an adhesive strip on their chest, capturing signals at a 250 Hz sample rate. The device recorded ECG voltage every 8 ms, estimating HR approximately every 10 s based on RR intervals, with an average of six HR estimates per minute. This device also measured other parameters such as HR variability (HRV). In this study, we focused on HR and tested HRV as part of sensitivity analyses. Cardiovascular data were transmitted via Bluetooth from the sensors to a dedicated study phone and securely uploaded to a server. Participants were instructed to wear the device 24 h a day for 5 consecutive days, only removing and replacing the strips when necessary (e.g., charging, during showers).

**HR**. Along with those 10‐s HR values, Preventice Inc. applied a proprietary algorithm that assigned reliability scores from 0% to 100%, with higher scores indicating greater reliability. As recommended, we only included HR values with a reliability score of 80% or higher in analyses. Since the EMA survey inquired about social interactions over the past 3 h, reliable HR data from that time frame were aggregated into mean values to align with the survey intervals, using the timestamps of both the survey and HR data. For example, HR data from 9 a.m. to 12 noon were averaged when caregivers completed the survey at noon. Given that stress triggers the “fight or flight” response, it leads to a sustained elevation in HR due to increased sympathetic nervous system activity.^29^ Thus, a higher‐than‐usual HR was expected to reflect an acute physiological indicator of stress.

In the sensitivity analysis, we tested HRV as an alternative measure of cardiovascular reactivity. HRV was calculated using the time‐domain index root mean squared successive differences (RMSSD), based on a 10‐s interval. This process involved first calculating the successive time differences between heartbeats (in milliseconds), squaring each difference, and then averaging the squared values before taking the square root of the total. Similar to HR analyses, reliable HRV data were aggregated into mean values to align with the survey intervals. Due to a skewed distribution, the average RMSSD scores at each 3‐h interval were log‐transformed prior to analysis. Moreover, greater HRV‐RMSSD is generally considered beneficial for health, while lower HRV (indicating reduced parasympathetic activity) may signal stress and is associated with poorer health outcomes, such as increased cardiovascular risk.^30^, ^31^, ^32^

### 2.4 Baseline interview covariates

We adjusted several covariates collected during the baseline interview, including **caregivers’ sociodemographics**: self‐reported race was coded as 1 (*non‐Hispanic Black*) and 0 (*non‐Hispanic White*), age (continuous variable in years), gender 1 (*female*) and 0 (*male*), highest level of education recoded as 1 (*college degree or above*) and 0 (*less than a college degree*), having any children 1 (*yes*) and 0 (*no*), marital status 1 (*currently married/living with a partner*) and 0 (*unmarried, including widowed, divorced, separated, or never married*), and employment status 1 (*currently employed full‐time or part‐time*) and 0 (*unemployed, including retired, student, stay‐at‐home, or on sick leave*). **Caregivers’ health conditions**: any heart‐related problems, including hypertension, heart problems, or stroke recoded as 1 (*yes*) and 0 (*no*), currently using heart‐related medication 1 (*yes*) and 0 (*no*), depressive symptoms (as a continuous variable) were assessed using the eight‐item Center for Epidemiologic Studies Depression scale (CESD) based on total scores,^33^ anxiety (as a continuous variable) was assessed based on the mean scores of four common anxiety symptoms: feeling nervous, tense, afraid, or worrying about everything.^34^ **Caregiving situations**: the relationship to the CR was dummy‐coded as spouse caregiver 1 (*yes*) and 0 (*no*), adult child caregiver 1 (*yes*) and 0 (*no*), and other caregivers 1 (*yes*) and 0 (*no*); years of caregiving (continuous variable in years); caregiver burden (a continuous variable) was assessed by 12‐item Zarit Burden^35^).

### 2.5 End of day covariates

We controlled for exercise level in our analyses to account for the extent to which the increases in HR were driven by greater physical activity. On a scale from 0 (*0 min*), 1 (*1*–*30 min*), 2 (*30*–*60 min*), 3 (*60*–*90 min*), 4 (*90*–*120 min*), to 5 (*more than 2 h*), participants reported daily time spent in moderate exercise and vigorous exercise, respectively, at the end of each day. To reduce model complexity (particularly in disentangling day‐level covariates), we converted both moderate and vigorous exercise into minutes using their respective midpoints (e.g., 0 = *0 min*, 1 = *15 min*, 2 = *45 min*, 3 = *75 min*, and so on). These two variables were then summed to create a single combined exercise variable representing the total minutes of exercise each study day. Participants also indicated the extent to which the day was typical for them, using a scale from 1 (*not at all*) to 5 (*a great deal*). Additionally, we controlled for whether the day was a weekday, coded as 1 (*yes*) and 0 (*no*).

### 2.6 Analytic strategy

Descriptive statistics were calculated by aggregating data at the *participant‐level*. Given the nested data structure, this study considered three‐level multilevel models, with assessments (*level‐1*) nested within days (*level‐2*) and participants (*level‐3*). Tests of the unconditional two‐level (assessment nested within participants) and three‐level models (assessment nested within days within participants) indicated that the three‐level model resulted in significantly better model fit (Δχ^2^[1]  =  42.67, *p*  < 0 .001), supporting the use of a three‐level model.

To obtain unbiased estimates for **research question 1** (**the within‐day effect of the amount of time and interaction quality with CRs on HR**), time‐varying predictors (i.e., the amount of time interacting with CRs and levels of positive and negative interaction with CRs) were partitioned into three levels using the centered within context techniques.^36^, ^37^, ^38^, ^39^ These included assessment‐level effects (*level‐1*; also within‐day effects), which reflect deviations of an individual assessment's raw score from the day‐level mean; day‐level effects (*level‐2;* also between‐day effects), which indicate deviations of the day‐level mean from the person‐specific mean; and person‐level effects (*level‐3;* also between‐person effects), represented by person‐specific means (i.e., each person's average score across all assessments during the study period). Day‐level covariates were similarly partitioned into day‐level and person‐level effects to account for variance at each level. Averaged HR every 3 h (*level‐1*) was treated as the continuous outcome. Of note, the number of surveys analyzed for levels of positive and negative interactions with CRs would be smaller than for the amount of time interacting with CRs, as caregivers did not report interacting with CRs in every survey.

Next, to test **research question 2 (the buffering effect of friend interactions and interaction quality with friends)**, interaction terms for friend variables (e.g., time interacting with CRs [*level 1*] × any friend interaction [*level 1*], time interacting with CRs [*level 1*] × any positive friend interaction [*level 1*], and time interacting with CRs [*level 1*] × any negative friend interaction [*level 1*]) were examined in separate models. We explored significant interactions with simple slope analysis.

Models were adjusted for covariates. All analyses focused on assessment‐level (*level‐1*) effects. Models included a random intercept, used Restricted Maximum Likelihood (REML) for fitting, and were performed in Stata 18 MP‐2.^40^

## 3 RESULTS

Table 1 presents sample characteristics. On average, caregivers completed 25.89 EMA surveys (SD = 5.67, range: 1–47). Approximately 80% of caregivers completed at least 24 EMA surveys (80% of the expected survey total). The average age of caregivers was 61.21, with a majority being non‐Hispanic White (63%), female (78%), having a college degree or higher (63%), married (61%), and having children (79%). About 39% were employed. More than three‐fifths of caregivers reported heart‐related health problems (63%), with 54% using heart medication. On average, caregivers had been providing care for 4.64 years. More than half were caring for a spouse, and 37% were providing care for aging parents. Daily experiences showed caregivers spent an average of 85 min interacting with CRs every 3 h. On average, caregivers reported interacting with friends in 16% of surveys, with positive friend interactions occurring in 15% of surveys and negative friend interactions in 8% of surveys. Caregivers had an average HR of 79.40 beats per minute and spent an average of 33.90 min per day exercising. On average, they rated the day as somewhat typical, leaning slightly toward being more typical than not (Mean_typicality_ = 3.15). Additionally, 67% of their survey days occurred on weekdays.

**TABLE 1:** Sample characteristics.

	Overall sample (*N *= 221)			
Parameter	Mean/%	SD	Minimum	Maximum
Race				
Non‐Hispanic White	63%			
Non‐Hispanic Black	37%			
Age	61.21	13.12	21.00	87.00
Female	78%	–	–	–
College degree or above	63%	–	–	–
Any children	79%	–	–	–
Married	61%	–	–	–
Employed	39%	–	–	–
Heart‐related problem	63%	–	–	–
Heart‐related medication	54%	–	–	–
Depression	7.30	5.23	0.00	23.00
Anxiety	2.01	0.67	1.00	4.00
Relationship types				
Spouse caregivers	51%	–	–	–
Adult child caregivers	37%	–	–	–
Other	12%	–	–	–
Years of caregiving	4.64	4.26	0.08	29.00
Caregiver burden	20.40	8.68	2.00	46.00
**EMA every 3‐h experiences**				
Time interacting with CR	84.96	36.02	1.00	177.39
Levels of positive interaction with CR	3.38	1.01	1.00	5.00
Levels of negative interaction with CR	1.79	0.68	1.00	4.56
Any friend interaction	0.16	0.17	0.00	0.77
Any positive friend interaction	0.15	0.16	0.00	0.77
Any negative friend interaction	0.08	0.11	0.00	0.62
Mean heart rate	79.40	10.91	55.12	123.92
Daily exercise (in min)	33.90	37.83	0.00	255.00
Typicality of day	3.15	0.75	1.2	5.00

Abbreviations: CR, care recipient; EMA, ecological momentary assessment; SD, standard deviation.

Overall, both unadjusted (without covariates) and adjusted (with covariates) models showed similar patterns. We focused on adjusted results in the main text as they provided more robust estimates by accounting for potential confounding variables. Unadjusted results were reported in the supplementary materials to show raw associations and allow comparison of changes in estimates.

### 3.1 Within‐day effects of CR time and interaction quality on HR among dementia caregivers

As shown in Table 2, adjusted multilevel models indicate that when caregivers had longer than usual interaction with CRs within a given day, their HR was higher during the same 3‐h period (*B *= 0.04, *p *< 0.001). Regarding interaction quality with CRs, adjusted multilevel models indicate that while levels of positive CR interaction did not link to HR (*B *= 0.31, *p *= 0.15), higher levels of CR negative interactions than usual within a given day were associated with higher HR during the same 3‐h period (*B *= 0.78, *p *< 0.001).

**TABLE 2:** Adjusted multilevel models examining the effects of time spent interacting with CRs, levels of positive interactions and negative interactions with CRs on HR.

	CR time		CR interaction quality	
Parameter	*B*	*SE*	*B*	*SE*
Intercept	91.69^***^	6.56	101.80^***^	8.70
**Assessment‐level (level‐1; within‐day effects)**				
Time with CR	0.04^***^	0.00	–	–
Levels of positive interaction with CR	–	–	0.31	0.21
Levels of negative interaction with CR	–	–	0.78^***^	0.19
**Day‐level (level‐2; between‐day effects)**				
Time with CR	−0.00	0.01	–	–
Levels of positive interaction with CR	–	–	−0.04	0.20
Levels of negative interaction with CR	–	–	0.01	0.20
**Person‐level (level‐3; between‐person effects)**				
Time with CR	0.02	0.02	–	–
Levels of positive interaction with CR	–	–	−1.54	1.19
Levels of negative interaction with CR	–	–	1.10	1.62
**Covariates**				
Black (vs. White)	2.06	1.54	1.86	1.54
Age	−0.30^***^	0.07	−0.34^***^	0.07
Female	1.60	1.67	1.32	1.71
College degree or above	−1.21	1.44	−0.96	1.45
Any children	2.32	1.86	1.82	1.88
Married	2.47	2.19	2.06	2.21
Employed	0.60	1.40	0.60	1.41
Heart‐related problem	−0.75	0.61	−1.03	0.61
Heart‐related medication	0.49	1.56	0.65	1.56
Depression	0.33	0.19	0.29	0.19
Anxiety	−0.10	1.42	−0.20	1.44
Child caregivers (vs. spouse)	1.55	2.44	1.41	2.44
Other caregivers (vs. spouse)	3.38	2.90	3.16	2.94
Years of caregiving	0.02	0.01	0.03	0.01
Caregiver burden	−0.09	0.10	−0.15	0.11
Physical exercise (level‐2)	0.03^***^	0.00	0.03^***^	0.01
Physical exercise (level‐3)	0.01	0.02	−0.00	0.02
Typicality of day	−0.58^***^	0.15	−0.70^***^	0.18
Weekday	0.07	0.29	−0.06	0.34
Observations	5167		3643	

*Note*: Time‐varying predictors were partitioned into three levels that is, centered within context. These included: assessment‐level effects (*level‐1*; also within‐day effects), which reflect deviations of an individual assessment's raw score from the day‐level mean; day‐level effects (*level‐2;* also between‐day effects), which indicate deviations of the day‐level mean from the person‐specific mean; and person‐level effects (*level‐3;* also between‐person effects), represented by person‐specific means (i.e., each person's average score across all assessments during the study period)

Abbreviations: CR, care recipient; HR, heart rate; SE, standard error.

*** *p* < 0.001.

### 3.2 The buffering effect of friend interactions in dementia caregivers

#### 3.2.1 Any presence of friend interaction as the moderator

**FIGURE 1:**
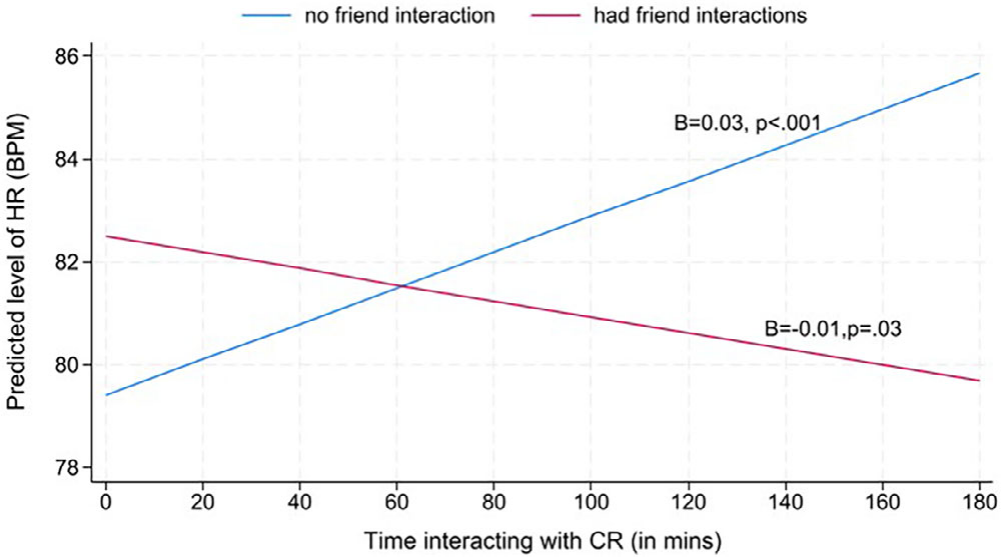
Within‐day effects of CR time on HR in dementia caregivers by any interaction with friends. When caregivers did not interact with friends (blue line), more CR interaction time was associated with increased HR. With friend interaction, more CR interaction time was linked to decreased HR (red line). CR, care recipient; HR, heart rate

As shown in Table 3, adjusted multilevel models revealed significant interaction effects of CR time (*level‐1*) × any friend interactions (*level‐1*) on HR (*B*_diff_ **= −0.05, *p *< 0.001). Simple slope analyses showed that when caregivers did not interact with friends, longer time interacting with CR than usual within a day was associated with increased HR (*B *= 0.03, *p *< 0.001). However, when caregivers interacted with friends, longer time interacting with CR than usual within a day was linked to lowered HR (*B *= −0.01; *p *= 0.03; Figure 1).

**TABLE 3:** Adjusted multilevel models examining the moderating effects of friend interactions on the effect of time interacting with CRs, levels of positive and negative interactions with CRs on HR.

	CR time		CR interaction quality	
Parameter	*B*	*SE*	*B*	*SE*
Intercept	91.70^***^	6.55	101.93^***^	8.71
**Assessment‐level (level‐1; within‐day effects)**				
Any friend interaction	3.09^***^	0.37	1.14^**^	0.37
CR time	0.03^***^	0.00	–	–
CR time × any friend interaction	−0.05^***^	0.01	–	–
CR positive interaction	–	–	0.39	0.21
CR negative interaction	–	–	0.88^***^	0.20
CR positive interaction × any friend interaction	–	–	−2.06^**^	0.72
CR negative interaction × any friend interaction	–	–	−1.50^*^	0.69
**Day‐level (level‐2; between‐day effects)**				
Any friend interaction	1.53	0.94	−0.10	1.11
CR time	−0.00	0.01		
CR positive interaction	–	–	−0.04	0.20
CR negative interaction	–	–	0.01	0.20
**Person‐level (level‐3; between‐person effects)**				
Any friend interaction	4.46	4.13	2.94	4.14
CR time	0.02	0.02		
CR positive interaction	–	–	−1.54	1.19
CR negative interaction	–	–	1.10	1.62
**Covariates**				
Black (vs. White)	2.08	1.54	1.92	1.54
Age	−0.30^***^	0.07	−0.34^***^	0.07
Female	1.41	1.70	1.08	1.73
College degree or above	−1.44	1.46	−1.20	1.47
Any children	2.39	1.86	1.89	1.88
Married	2.49	2.19	2.14	2.20
Employed	0.55	1.40	0.58	1.41
Heart‐related problem	−0.73	0.61	−1.02	0.61
Heart‐related medication	0.49	1.56	0.68	1.56
Depression	0.33	0.19	0.29	0.19
Anxiety	−0.01	1.42	−0.10	1.44
Child caregivers (vs. spouse)	1.52	2.43	1.35	2.44
Other caregivers (vs. spouse)	3.21	2.91	2.98	2.95
Years of caregiving	0.02	0.01	0.02	0.01
Caregiver burden	−0.09	0.10	−0.15	0.11
Physical exercise (level‐2)	0.03^***^	0.00	0.03^***^	0.01
Physical exercise (level‐3)	0.00	0.02	−0.01	0.02
Typicality of day	−0.57^***^	0.15	−0.70^***^	0.18
Weekday	0.08	0.29	−0.04	0.34
Observations	5167		3643	

*Note*: Time‐varying predictors were partitioned into three levels that is, centered within context. These included: assessment‐level effects (*level‐1*; also within‐day effects), which reflect deviations of an individual assessment's raw score from the day‐level mean; day‐level effects (*level‐2;* also between‐day effects), which indicate deviations of the day‐level mean from the person‐specific mean; and person‐level effects (*level‐3;* also between‐person effects), represented by person‐specific means (i.e., each person's average score across all assessments during the study period).

Abbreviations: CR, care recipient; HR, heart rate; SE, standard error.

^*^ *p *< 0.05;

^**^ *p* < 0.01;

^***^ *p* < 0.001.

**FIGURE 2:**
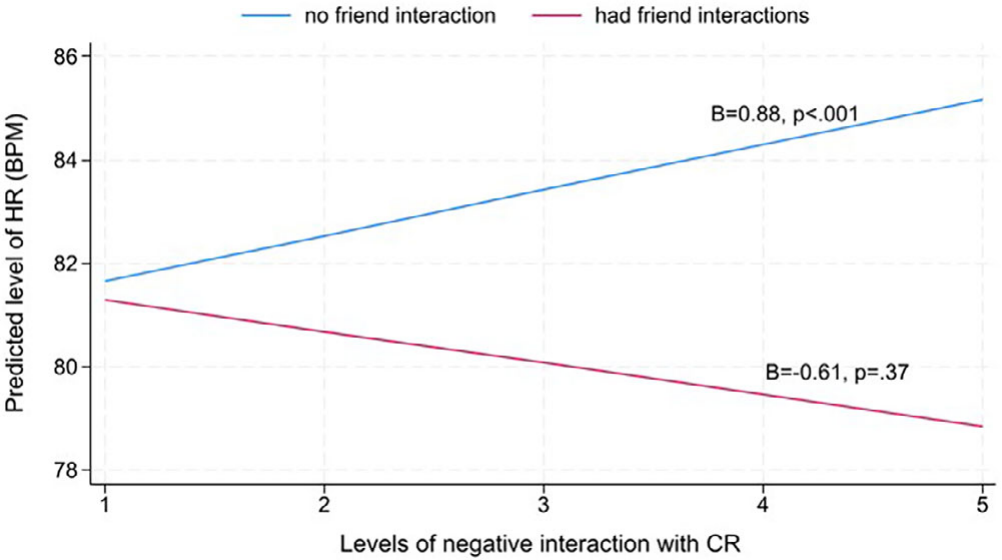
Within‐day effects of levels of negative interaction with CR on heart rate in dementia caregivers by any interaction with friends. Higher levels of negative interactions with CRs were linked to increased HR when caregivers did not interact with friends (blue line). However, when caregivers interacted with friends, higher levels of negative interactions with CRs were not associated with HR (red line). CR, care recipient; HR, heart rate

Regarding interaction quality with CRs, adjusted multilevel models revealed a significant interaction effect of CR negative interaction (*level‐1*) × any friend interaction (*level‐1*) on HR (*B*_diff_ = −1.50, *p *= 0.03; Table 3). Simple slope analysis indicated that within a day, higher levels of negative interactions with CRs were linked to increased HR when caregivers did not interact with friends (*B *= 0.88; *p *< 0.001); however, when caregivers interacted with friends, higher levels of negative interactions with CRs were not linked to HR (*B *= −0.61, *p = 0*.37; Figure 2).

**FIGURE 3:**
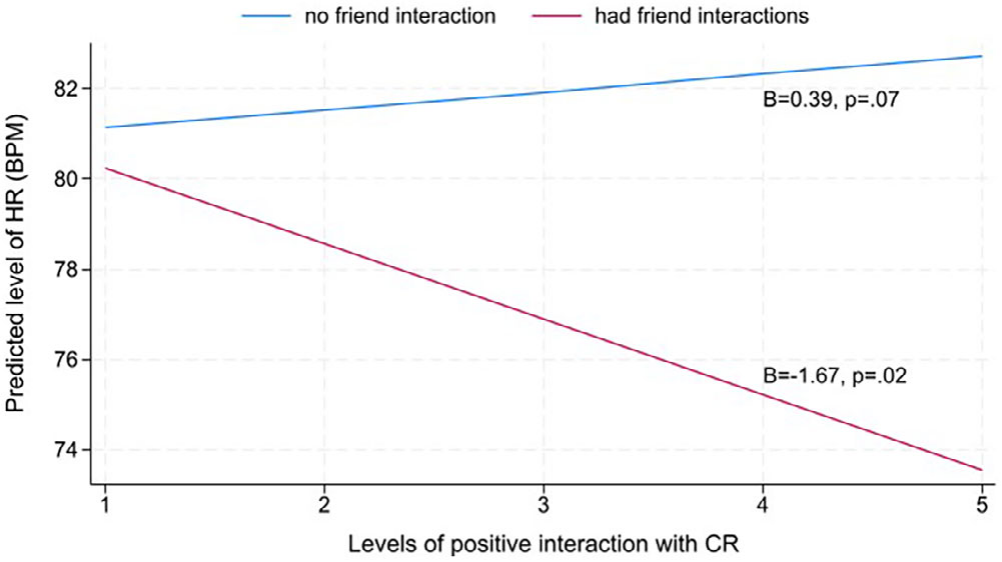
Within‐day effects of levels of positive interaction with CR on heart rate in dementia caregivers by any interaction with friends. Higher levels of positive interactions with CRs were linked to reduced HR when caregivers also interacted with friends (red line). When caregivers did not interact with friends, higher levels of positive interactions with CRs were not associated with HR (blue line). CR, care recipient; HR, heart rate

The interaction terms of CR positive interaction (*level‐1*) × any friend interaction (*level‐1*) on HR were also significant (*B*_diff_ = −2.06, *p *= 0.004; Table 3). Simple slope analysis indicated that, within a day, higher levels of positive interactions with CRs were linked to reduced HR only when caregivers also interacted with friends (*B *= −1.67; *p *= 0.02). When caregivers did not interact with friends, higher levels of positive interactions with CRs were not linked to HR (*B *= 0.39, *p *= 0.07; Figure 3).

#### 3.2.2 Any positive and any negative friend interaction as moderators

We examined the quality of interaction with friends, analyzing any positive and any negative interaction with friends in each 3‐h interval as separate binary moderators. As shown in Table 4, results using these binary variables were generally consistent with those obtained when considering any presence of friend interactions. Specifically, with regard to time interacting with CR as the predictor, positive interactions with friends moderate the impact of time interacting with CRs on HR (*B*_diff_ = −0.05, *p *< 0.001). Interestingly, negative interactions with friends also demonstrated a similar buffering effect (*B*_diff_ = −0.05, *p *< 0.001). Simple slope analyses revealed the same patterns of findings as in Figure 3 (see Figures S1 and S2).

**TABLE 4:** Adjusted multilevel models examining the moderating effects of any positive and any negative friend interactions on the effect of time interacting with CR, levels of positive and negative interactions with CRs on HR.

	Moderating effect of positive interaction with friends				Moderating effect of negative interaction with friends			
Parameter	*B*	*SE*	*B*	*SE*	*B*	*SE*	*B*	*SE*
Intercept	91.69^***^	6.54	102.00^***^	8.69	91.90^***^	6.52	101.89^***^	8.67
**Assessment‐level (level‐1; within‐day effects)**								
Any positive friend interaction	3.09^***^	0.38	0.97^**^	0.37	–	–	–	–
Any negative friend interaction	–	–	–	–	3.07^***^	0.48	1.03^*^	0.47
CR time	0.04^***^	0.00	–	–	0.04^***^	0.00	–	–
CR positive interaction	–	–	0.36	0.21	–	–	0.36	0.21
CR negative interaction	–	–	0.85^***^	0.20	–	–	0.85^***^	0.19
CR time × any positive friend interaction	−0.05^***^	0.01	–	–	–	–	–	–
CR positive interaction × any positive friend interaction	–	–	−1.59^*^	0.73	–	–	–	–
CR negative interaction × any positive friend interaction	–	–	−1.05	0.69	–	–		
CR time × any negative friend interaction	–	–	–	–	−0.05^***^	0.01	–	–
CR positive interaction × any negative friend interaction	–	–	–	–	–	–	−2.19^*^	0.93
CR negative interaction × any negative friend interaction	–	–	–	–	–	–	−1.40	0.83
**Day‐level (level‐2; between‐day effects)**								
Any positive friend interaction	1.87	0.98	0.36	1.16				
Any negative friend interaction	–	–	–	–	1.64	1.23	0.39	1.44
CR time	−0.00	0.01	–	–	−0.00	0.01	–	–
CR positive interaction	–	–	−0.04	0.20	–	–	−0.04	0.20
CR negative interaction	–	–	0.01	0.20	–	–	0.01	0.20
**Person‐level (level‐3; between‐person effects)**								
Any positive friend interaction	5.95	4.39	4.37	4.40	–	–	–	–
Any negative friend interaction	–	–	–	–	10.27	6.28	8.16	6.29
CR time	0.02	0.02	–	–	0.02	0.02	–	–
CR positive interaction	–	–	−1.56	1.18	–	–	−1.49	1.18
CR negative interaction	–	–	1.07	1.62	–	–	1.05	1.62
Black (vs. White)	2.10	1.53	1.94	1.54	1.91	1.53	1.75	1.54
Age	−0.30^***^	0.07	−0.34^***^	0.07	−0.31^***^	0.07	−0.35^***^	0.07
Female	1.32	1.69	1.01	1.72	1.66	1.67	1.35	1.70
College degree or above	−1.48	1.45	−1.23	1.46	−1.47	1.44	−1.22	1.45
Any children	2.46	1.86	1.94	1.88	2.43	1.85	1.93	1.87
Married	2.48	2.19	2.15	2.20	2.22	2.19	1.91	2.20
Employed	0.56	1.40	0.58	1.41	0.72	1.40	0.73	1.41
Heart‐related problem	−0.73	0.61	−1.01	0.61	−0.78	0.60	−1.07	0.61
Heart‐related medication	0.53	1.56	0.70	1.56	0.61	1.55	0.75	1.56
Depression	0.33	0.19	0.29	0.19	0.33	0.19	0.29	0.19
Anxiety	0.01	1.42	−0.07	1.44	0.06	1.42	−0.07	1.43
Child caregivers (vs. spouse)	1.52	2.43	1.37	2.44	1.47	2.42	1.34	2.43
Other caregivers (vs. spouse)	3.16	2.90	2.94	2.95	3.15	2.89	2.96	2.93
Years of caregiving	0.02	0.01	0.02	0.01	0.02	0.01	0.02	0.01
Caregiver burden	−0.09	0.10	−0.15	0.11	−0.10	0.10	−0.16	0.11
Physical exercise (level‐2)	0.03^***^	0.00	0.03^***^	0.01	0.03^***^	0.00	0.03^***^	0.01
Physical exercise (level‐3)	0.00	0.02	−0.01	0.02	0.00	0.02	−0.01	0.02
Typicality of day	−0.55^***^	0.15	−0.69^***^	0.18	−0.56^***^	0.15	−0.69^***^	0.18
Weekday	0.08	0.29	−0.04	0.34	0.09	0.29	−0.03	0.34
Observations	5167		3643		5167		3643	

*Note*: Time‐varying predictors were partitioned into three levels that is, centered within context. These included: assessment‐level effects (*level‐1*; also within‐day effects), which reflect deviations of an individual assessment's raw score from the day‐level mean; day‐level effects (*level‐2;* also between‐day effects), which indicate deviations of the day‐level mean from the person‐specific mean; and person‐level effects (*level‐3;* also between‐person effects), represented by person‐specific means (i.e., each person's average score across all assessments during the study period).

Abbreviations: CR, care recipient; HR, heart rate; SE, standard error.

* *p *< 0.05;

** *p *< 0.01;

*** *p *< 0.001.

Regarding models involving predictors of interaction quality with CR, CR positive interactions × any positive friend interaction (*B*_diff_ = −1.59, *p *= 0.03), and CR positive interactions × any negative friend interaction (*B*_diff_ = ‐2.19, *p *= 0.02) were significant as well. Simple slope analyses revealed a trend that higher levels of positive interactions with CRs were linked to lowered HR when caregivers experienced either positive or negative interactions with friends during the same 3‐h interval (similar patterns of simple slope findings as in Figure 3; see Figures S3 and S4). However, interaction quality with friends did not moderate the association between CR negative interactions and HR. The interaction terms, CR negative interactions × any positive friend interaction (*B*_diff_ = −1.05, *p *= 0.13) and CR negative interactions × any negative friend interaction (*B*_diff_ = −1.40, *p *= 0.09), were non‐significant.

### 3.3 Sensitivity tests

Sensitivity tests were conducted to verify the robustness of the findings. Unadjusted models (without covariates) showed similar patterns of results (see Tables S1 and S2), suggesting the robustness of the findings. Moreover, HRV (log transformed HRV‐RMSSD) was tested as an alternative outcome. In adjusted models, time interacting with CR was associated with reduced HRV, indicating acute stress or poor cardiovascular health in dementia caregivers, with this effect primarily driven by negative interactions (Table S3). However, friend interaction (or the quality of their interaction; results not shown) did not moderate the link between caregiving (either CR time or CR interaction quality) and HRV (Table S4).

We also assessed the direct link between interaction with friends and HR (i.e., whether interactions with friends have a direct momentary effect on HR, regardless of whether CR was present). Finding revealed that when caregivers had interactions with friends within a given day, their HR was higher during the same 3‐h period (*B *= 4.20, *p *< 0.001; Table S5), which may reflect mild physiological arousal due to social engagement.

We also examined whether interactions with anyone, interactions with family (including spouse, children, parents, siblings, grandchildren, grandparents, and other family members), or interactions with other social partners (including neighbors, service providers, coworkers, acquaintances, and strangers) moderated the within‐day effect of time spent interacting with CR on HR. Adjusted multilevel models revealed significant interactions between CR time (*level*‐1) and any social interactions (*level*‐1) on HR (*B*_diff_ **= −0.06, *p *< 0.001). Without social interactions, more CR time was linked to increased HR (*B *= 0.02, *p *< 0.001), but with social interactions, more CR time was associated with lower HR (*B *= −0.04, *p *< 0.001). This effect was primarily driven by family and friends, as testing family interactions as the moderator (*B*_diff_ = −0.06, *p *< 0.001) yielded a similar pattern of findings as friend interactions. When caregivers had no family interaction, more CR time was linked to higher HR (*B *= 0.02, *p *< 0.001); with family interaction, more CR time was associated with lower HR (*B *= −0.03, *p *< 0.001). We found a significant interaction between CR time and other social partner interactions (*B*_diff_ = −0.04, *p *< 0.001). However, when caregivers interacted with other social partners, CR time was not significantly associated with HR (*B *= −0.01, *p *= 0.49). In contrast, when caregivers did not interact with others, more CR time was linked to higher HR (*B *= 0.04, *p *< 0.001).

## 4 DISCUSSION

Few studies have investigated the daily experiences of dementia caregivers.^3^, ^41^, ^42^ Within this limited body of research, most have focused on caregiving stress and subjective emotional outcomes. This study advances the field by examining dementia caregivers’ daily interactions with their CRs and assessing fluctuations in HR in the moment through a 5‐day EMA study with a diverse group of caregivers. It also innovates by examining the unique stress‐buffering role of friends in caregiving contexts, responding to a call for scholarly attention to “Friendship and Caregiving.”^12^ These daily patterns are critical for understanding how caregiving‐related and friendship experiences accumulate over time, shaping long‐term cardiovascular health and the progression of related diseases.

### 4.1 Within‐day effects of CR time and interaction quality on HR among dementia caregivers

Our results indicated that for every additional minute in contact with a CR, there was a 0.04‐unit increase in HR. Over the 3‐h assessment period, this could equate to a meaningful 7.2‐unit increase in HR from interacting with the CR versus not interacting with the CR within the same caregiver. This highlights the heightened physiological reactivity of extended time interacting with CRs. Notably, while momentary increases in HR (acute) are not necessarily harmful if they return to normal quickly, chronic elevations in HR over prolonged periods could impose greater cardiovascular strain, which in turn increases the risk of adverse health outcomes.^43^ Moreover, longer contact with CRs not only may induce psychosocial stress (e.g., being overwhelmed or frustrated with caregiving) but also may link to physical stress (e.g., physical exertion from lifting or assisting with mobility, fatigue, and sleep deprivation).^44^, ^45^ Both forms of stress can contribute to elevated HR.

Our findings on interaction quality with CRs provide additional insights. Negative interactions with CRs emerged as a stronger factor of elevated HR, consistent with Cannon's fight‐or‐flight stress response theory.^46^, ^47^ According to this theory, the human body's stress response is automatically activated in reactions to perceived threats and stressful situations. Greater levels of negative interactions with CRs, such as CR's behavior problems (e.g., aggression), requiring help with personal tasks like bathing and toileting, or asking repetitive questions due to memory impairments,^48^, ^49^, ^50^ may be perceived as stressors by caregivers, leading to heightened physiological responses. In contrast, positive interactions with CRs are often perceived as supportive, which may reduce stress and cardiovascular reactivity. Overall, findings indicate that negative daily interactions with CRs are more salient than positive interactions in contributing to caregivers’ cardiovascular strain.

### 4.2 The buffering effect of friend interactions in dementia caregivers

In this study, we investigated a potentially modifiable social factor influencing caregivers’ cardiovascular health. Findings suggest that the effect of longer time and higher levels of negative interactions with CRs on increased HR can be attenuated by interactions with friends. This aligns with prior literature suggesting friends have the potential to alleviate some of the stress associated with caregiving.^14^ Interactions with friends generally involve positive daily experiences unrelated to caregiving (e.g., emotional support, companionship, shared leisure activities), and thus, simply connecting with them—regardless of the mode of contact—may help counterbalance caregiving‐related stress.^27^, ^51^ Alternatively, some friends may support dementia caregivers by directly involving themselves in caregiving (e.g., providing in‐home respite assistance),^16^ allowing caregivers to recharge and relax, which may reduce stress and improve well‐being. Moreover, caregivers may face challenges in maintaining relationships outside the caregiver–CR dyad due to limited time and reduced opportunities to socialize (e.g., being unable to go out with friends).^15^, ^52^ As such, interactions with friends can serve as a source of social normalcy, helping to reduce stress and mitigate caregiving‐related HR elevations. While friend interactions may reduce HR during negative caregiving moments, sensitivity tests revealed that friend interactions alone (without considering the amount of time or interaction quality with CRs) were temporarily associated with increased HR. This may reflect mild physiological arousal due to social engagement. Given this, the physiological impact of social interactions on caregivers may be more complex and warrants further investigation.

Positive CR interactions alone are not associated with lower HR. Yet, they were associated with lower HR when caregivers also interacted with friends within the same 3‐h period, indicating a lower or non‐stress response. Supporting this, a laboratory study by Uchino et al.^53^ found that caregivers who had positive and closer emotional bonds with their CRs exhibited lower baseline HR. In addition to improving caregiver health outcomes, studies have shown that caregiver emotional closeness with CRs—a potentially modifiable factor—was associated with better CR cognitive outcomes and lower informal dementia care costs.^54^, ^55^

Regarding interaction quality with friends, findings support the hypothesis that positive interactions with friends buffer the impact of longer interactions with CRs on elevated HR. Surprisingly, negative friend interactions also show a buffering effect. These findings may indicate that the mere act of socializing with friends may be beneficial, even if the interactions are not always positive. Research suggests that friendships across adulthood are generally perceived as positive^27^ and may foster a sense of social connectedness; therefore, it is possible that the overall positive effects of friendships may outweigh the temporary negative interaction (e.g., feeling upset about being unable to make plans with friends).^56^ Alternatively, friend interactions (regardless of positive or negative) might serve as a distraction from caregiving‐related stressors, temporarily alleviating physiological stress responses associated with caregiving. Although both positive and negative interactions with friends show similar cardiovascular reactivity, this does not mean the quality of friend interactions is unimportant for health. Other aspects of health (e.g., emotional reactivity) may still be differently influenced by interaction quality with friends.

Comparisons of the buffering effects of friends, family, and others were not the focus of this study; however, findings from sensitivity tests suggest that social interactions in general help buffer the impact of CR time on HR, with interactions with friends and family being particularly associated with reduced HR during extended CR time. This is consistent with studies indicating that friends and family each play distinct supportive roles for dementia caregivers.^10^ For example, among spousal dementia caregivers, friends with similar caregiving experiences may offer optimal emotional support, while adult children may provide tangible assistance and positive social interaction, which in turn alleviates stress‐related physiological responses.

Together, these findings highlight the importance of reducing negative exchanges within the caregiver–CR dyad and fostering more friend interactions to enhance daily cardiovascular health. Friend‐based interventions addressing the social and emotional needs of dementia caregivers could significantly improve cardiovascular outcomes. For example, friendly visitor and befriending programs (e.g., weekly or monthly visits with a peer volunteer, designed to foster a friendship with the caregiver) may help reduce social isolation and improve perceived social support, depression, and loneliness,^57^, ^58^, ^59^, ^60^ thereby enhancing cardiovascular health.

### 4.3 Limitations and future directions

This study has several limitations that warrant consideration. First, we did not examine detailed information about interactions with CRs besides time allocation and interaction quality. Future studies that integrate qualitative reports or data about caregivers’ activities during their interaction with CR (e.g., jogging with CRs, lifting CRs into bed) could provide deeper insights into how these daily experiences with CR and physiological reactivity align. Similarly, additional studies could examine nuances of caregivers’ interactions with friends (e.g., activity engagements, support exchanges), along with the mode of interactions (e.g., in‐person vs. technologically mediated interactions), and the preexisting nature of these friendships (e.g., shared friendships between caregivers and CRs, long‐term friendships) to better understand how daily interaction with friends alleviates caregiving stress. Moreover, our study did not directly focus on caregiving provided by friends. Future studies should address questions regarding the willingness and motivation to provide intensive caregiving to friends with dementia as well as the efficacy of friend caregiving.^12^

While we controlled for exercise at the daily level, future research should collect other proximal contextual factors such as smoking, alcohol consumption, speaking (e.g., engaging in conversations), and location (e.g., being at home vs. at work vs. in public settings), as these factors may be temporally tied to HR measurements, and controlling for them could offer a more comprehensive understanding of cardiovascular reactivity in daily settings ^61^, ^62^, ^63^, ^64^. Last but not least, the sample size of the study was insufficient for stratified analyses of caregiver–CR relationships (e.g., spousal vs. adult child caregivers), which may influence the observed associations. Future research should explore how relationship factors with CRs (e.g., relationship types and relationship closeness) affect caregiving experiences and cardiovascular outcomes.

In conclusion, this study revealed that spending more time interacting with CRs than usual within a day was linked to increased HR, primarily driven by negative interactions with CRs; yet, friend interactions can temporarily buffer this adverse effect. When positive CR interactions were paired with friend interactions, they were associated with lower HR. Additionally, the findings suggest that simply socializing with friends can be beneficial, even if the interactions occasionally involve negativity. If these findings are replicated in larger studies, interventions could focus on reducing negative interactions within the caregiver–CR dyad and promoting more friend interactions for better cardiovascular health among dementia caregivers.

## CONFLICT OF INTEREST STATEMENT

All authors and co‐authors have no conflicts of interest to declare. Author disclosures are available in the supporting information.

## CONSENT STATEMENT

All human subjects provided informed consent.

Human studies

## ETHICS REVIEW

The study was approved by the University of Michigan Ethics Review Board and was performed in accordance with the ethical standards as laid down in the 1964 Declaration of Helsinki and its later amendments or comparable ethical standards.

## STATEMENT ADDRESSING DIVERSITY, EQUITY, AND INCLUSION (DEI)

This study recruited a diverse group of dementia caregivers, including Black and White participants to enhance generalizability across racial groups. This study also included caregivers of individuals with dementia who had not received a formal diagnosis, addressing the lower rates of dementia diagnoses among Black Americans.

## Supporting information



Supporting Information

Supporting Information
