# Supplementary material for: Daily interactions with care recipients and cardiovascular reactivity among dementia caregivers: The buffering role of friend interactions
Source: Alzheimers Dement. 2025 May 15;21(5):e70281. doi: 10.1002/alz.70281 (PMC12079534; doi:10.1002/alz.70281)
Supplement: Supplementary file 1 — Supporting Information [file ALZ-21-e70281-s001.docx]

Supplementary Table 1.
Unadjusted Multilevel Models Examining the Effects of Time Spent with Care Recipients, Positive Interactions, Negative Interactions on Heart Rate (HR)

|  | CR Time | | |  |  | Interaction quality | | |  |
| --- | --- | --- | --- | --- | --- | --- | --- | --- | --- |
|  | *B* |  | *SE* |  |  | *B* |  | *SE* |  |
| Intercept | 81.14 | *** | 1.85 |  |  | 86.34 | *** | 6.14 |  |
| **Assessment-level (level-1; within-day effects)** |  |  |  |  |  |  |  |  |  |
| CR Time | 0.04 | *** | 0.00 |  |  | – |  | – |  |
| CR Positive interaction | – |  | – |  |  | 0.32 |  | 0.21 |  |
| CR Negative interaction | – |  | – |  |  | 0.86 | *** | 0.19 |  |
| **Day-level (level-2; between-day effects)** |  |  |  |  |  |  |  |  |  |
| CR T-me | 0.00 |  | 0.01 |  |  | – |  | – |  |
| CR Positive interaction | – |  | – |  |  | -0.09 |  | 0.20 |  |
| CR Negative interaction | – |  | – |  |  | 0.11 |  | 0.20 |  |
| **Person-level (level-3; between-person effects)** |  |  |  |  |  |  |  |  |  |
| CR Time | -0.02 |  | 0.02 |  |  | – |  | – |  |
| CR Positive interaction | – |  | – |  |  | -0.95 |  | 1.24 |  |
| CR Negative interaction | – |  | – |  |  | -0.31 |  | 1.64 |  |
| Observations | 5589 | | |  |  | 3888 | | |  |

Notes: Time-varying predictors were partitioned into three levels i.e., centered within context. These included: assessment-level effects (*level-1*; also within-day effects), which reflect deviations of an individual assessment’s raw score from the day-level mean; day-level effects (*level-2;* also between-day effects), which indicate deviations of the day-level mean from the person-specific mean; and person-level effects (*level-3;* also between-person effects), represented by person-specific means (i.e., each person’s average score across all assessments during the study period).

*** *p*<.001.

Supplementary Table 2.
Unadjusted Multilevel Models Examining the Moderating Effects of Friend Interactions on the Effect of Time Spent with Care Recipients, Positive Interactions, Negative Interactions on Heart Rate (HR)

|  | CR Time | | |  |  | Interaction Quality | | |  |
| --- | --- | --- | --- | --- | --- | --- | --- | --- | --- |
|  | *B* |  | *SE* |  |  | *B* |  | *SE* |  |
| Intercept | 81.19 | *** | 1.94 |  |  | 86.26 | *** | 6.16 |  |
| **Assessment-level (level-1; within-day effects)** |  |  |  |  |  |  |  |  |  |
| Friend interaction | 3.04 | *** | 0.36 |  |  | 1.12 | ** | 0.36 |  |
| CR Time | 0.03 | *** | 0.00 |  |  | – |  | – |  |
| CR Time × Friend interaction | -0.05 | *** | 0.01 |  |  | – |  | – |  |
| CR Positive interaction | – |  | – |  |  | 0.39 |  | 0.21 |  |
| CR Negative interaction | – |  | – |  |  | 0.95 | *** | 0.19 |  |
| CR Positive interaction × Friend interaction | – |  | – |  |  | -1.74 | * | 0.72 |  |
| CR Negative interaction × Friend interaction | – |  | – |  |  | -1.29 |  | 0.68 |  |
| **Day-level (level-2; between-day effects)** |  |  |  |  |  |  |  |  |  |
| Friend interaction | 1.42 |  | 0.95 |  |  | -0.32 |  | 1.09 |  |
| CR Time | 0.00 |  | 0.01 |  |  | – |  | – |  |
| CR Positive interaction | – |  | – |  |  | -0.09 |  | 0.20 |  |
| CR Negative interaction | – |  | – |  |  | 0.11 |  | 0.20 |  |
| **Person-level (level-3; between-person effects)** |  |  |  |  |  |  |  |  |  |
| Friend interaction | 0.31 |  | 4.27 |  |  | -0.68 |  | 4.36 |  |
| CR Time | -0.02 |  | 0.02 |  |  | – |  | – |  |
| CR Positive interaction | – |  | – |  |  | -0.92 |  | 1.24 |  |
| CR Negative interaction | – |  | – |  |  | -0.29 |  | 1.64 |  |
| Observations | 5589 | | |  |  | 3888 | | |  |

Notes: Time-varying predictors were partitioned into three levels i.e., centered within context. These included: assessment-level effects (*level-1*; also within-day effects), which reflect deviations of an individual assessment’s raw score from the day-level mean; day-level effects (*level-2;* also between-day effects), which indicate deviations of the day-level mean from the person-specific mean; and person-level effects (*level-3;* also between-person effects), represented by person-specific means (i.e., each person’s average score across all assessments during the study period).

**p* <.05; ***p* <.01; *** *p*<.001.

|  | CR Time | | |  |  | Interaction quality | | |  |
| --- | --- | --- | --- | --- | --- | --- | --- | --- | --- |
|  | *B* |  | *SE* |  |  | *B* |  | *SE* |  |
| Intercept | 3.30 | *** | 0.41 |  |  | 3.16 | *** | 0.54 |  |
| **Assessment-level (level-1; within-day effects)** |  |  |  |  |  |  |  |  |  |
| CR Time (in hours) | -0.05 | *** | 0.00 |  |  | – |  | – |  |
| CR Positive interaction | – |  | – |  |  | -0.00 |  | 0.01 |  |
| CR Negative interaction | – |  | – |  |  | -0.02 | ** | 0.01 |  |
| **Day-level (level-2; between-day effects)** |  |  |  |  |  |  |  |  |  |
| CR Time (in hours) | -0.01 |  | 0.02 |  |  | – |  | – |  |
| CR Positive interaction | – |  | – |  |  | -0.01 |  | 0.02 |  |
| CR Negative interaction | – |  | – |  |  | 0.01 |  | 0.02 |  |
| **Person-level (level-3; between-person effects)** |  |  |  |  |  |  |  |  |  |
| CR Time (in hours) | -0.07 |  | 0.08 |  |  | – |  | – |  |
| CR Positive interaction | – |  | – |  |  | 0.01 |  | 0.07 |  |
| CR Negative interaction | – |  | – |  |  | -0.12 |  | 0.10 |  |
| Covariates |  |  |  |  |  |  |  |  |  |
| Observations | 5172 | | |  |  | 3645 | | |  |

Supplementary Table 3.
Adjusted Multilevel Models Examining the Effects of Time Spent with Care Recipients, Levels of Positive and Negative Interactions with CRs on Log Transformed Heart Rate Variability (HRV-RMSSD)

Notes: Time-varying predictors were partitioned into three levels i.e., centered within context. These included: assessment-level effects (*level-1*; also within-day effects), which reflect deviations of an individual assessment’s raw score from the day-level mean; day-level effects (*level-2;* also between-day effects), which indicate deviations of the day-level mean from the person-specific mean; and person-level effects (*level-3;* also between-person effects), represented by person-specific means (i.e., each person’s average score across all assessments during the study period). Covariates included caregiver’s race (if in full sample model), age, gender, education level (college degree or above), parental status (any children), marital status, employment status, heart-related problems or medication, depression, anxiety, relationship type, years of caregiving, caregiver burden, daily physical activity level, typicality of the study day, and whether the study day was a weekday.

**p* <.05; ***p* <.01; *** *p*<.001.

Supplementary Table 4.
Adjusted Multilevel Models Examining the Moderating Effects of Any Friend Interactions on the Effect of Time Spent with Care Recipients, Levels of Positive and Negative Interactions with CRs on Log Transformed Heart Rate Variability (HRV-RMSSD)

|  | CR Time | | |  |  | Interaction quality | | |  |
| --- | --- | --- | --- | --- | --- | --- | --- | --- | --- |
|  | *B* |  | *SE* |  |  | *B* |  | *SE* |  |
| Intercept | 3.31 | *** | 0.41 |  |  | 3.17 | *** | 0.54 |  |
| **Assessment-level (level-1; within-day effects)** |  |  |  |  |  |  |  |  |  |
| Any friend interaction | -0.08 | *** | 0.02 |  |  | -0.05 | ** | 0.02 |  |
| CR Time in hours | -0.05 | *** | 0.00 |  |  | – |  | – |  |
| CR Time in hours × Any friend interaction | 0.03 |  | 0.02 |  |  | – |  | – |  |
| CR Positive interaction | – |  | – |  |  | 0.00 |  | 0.01 |  |
| CR Negative interaction | – |  | – |  |  | -0.02 | ** | 0.01 |  |
| CR Positive interaction × Any friend interaction | – |  | – |  |  | -0.02 |  | 0.03 |  |
| CR Negative interaction × Any friend interaction | – |  | – |  |  | -0.02 |  | 0.03 |  |
| **Day-level (level-2; between-day effects)** |  |  |  |  |  |  |  |  |  |
| Friend interaction | 0.00 |  | 0.04 |  |  | 0.03 |  | 0.05 |  |
| CR Time in hours | -0.01 |  | 0.02 |  |  | – |  | – |  |
| CR Positive interaction | – |  | – |  |  | -0.02 |  | 0.02 |  |
| CR Negative interaction | – |  | – |  |  | 0.01 |  | 0.02 |  |
| **Person-level (level-3; between-person effects)** |  |  |  |  |  |  |  |  |  |
| Friend interaction | 0.17 |  | 0.26 |  |  | 0.18 |  | 0.26 |  |
| CR Time in hours | -0.08 |  | 0.08 |  |  | – |  | – |  |
| CR Positive interaction | – |  | – |  |  | 0.00 |  | 0.07 |  |
| CR Negative interaction | – |  | – |  |  | -0.12 |  | 0.10 |  |
| Covariates |  |  |  |  |  |  |  |  |  |
| Observations | 5172 | | |  |  | 3645 | | |  |

Notes: Time-varying predictors were partitioned into three levels i.e., centered within context. These included: assessment-level effects (*level-1*; also within-day effects), which reflect deviations of an individual assessment’s raw score from the day-level mean; day-level effects (*level-2;* also between-day effects), which indicate deviations of the day-level mean from the person-specific mean; and person-level effects (*level-3;* also between-person effects), represented by person-specific means (i.e., each person’s average score across all assessments during the study period). Covariates included caregiver’s race (if in full sample model), age, gender, education level (college degree or above), parental status (any children), marital status, employment status, heart-related problems or medication, depression, anxiety, relationship type, years of caregiving, caregiver burden, daily physical activity level, typicality of the study day, and whether the study day was a weekday.

**p* <.05; ***p* <.01; *** *p*<.001.

Supplementary Table 5.

Adjusted Multilevel Models Examining the Main Effects of Any Friend Interactions on Heart Rate (HR)

|  | *B* |  | *SE* |
| --- | --- | --- | --- |
| Intercept | 93.19 | *** | 6.27 |
| Any friend interaction (level-1; within-day effects) | 4.20 | *** | 0.38 |
| Any friend interaction (level-2; between-day effects) | 0.70 |  | 0.93 |
| Any friend interaction (level-3; between-person effects) | 3.55 |  | 4.12 |
| Covariates |  |  |  |
| Black (vs White) | 1.96 |  | 1.53 |
| Age | -0.30 | *** | 0.07 |
| Female | 1.53 |  | 1.68 |
| College degree or above | -1.60 |  | 1.45 |
| Any children | 2.61 |  | 1.85 |
| Married | 2.27 |  | 2.17 |
| Employed | 0.50 |  | 1.40 |
| Heart-related problem | -0.73 |  | 0.61 |
| Heart-related medication | 0.42 |  | 1.55 |
| Depression | 0.34 |  | 0.19 |
| Anxiety | 0.01 |  | 1.42 |
| *Child caregivers (vs spouse)* | 1.13 |  | 2.37 |
| *Other caregivers (vs spouse)* | 2.82 |  | 2.85 |
| Years of caregiving | 0.02 |  | 0.01 |
| Caregiver burden | -0.10 |  | 0.10 |
| Physical exercise (level-2) | 0.03 | *** | 0.00 |
| Physical exercise (level-3) | 0.00 |  | 0.02 |
| Typicality of day | -0.58 | *** | 0.15 |
| Weekday | 0.09 |  | 0.29 |
| Observations |  | 5167 |  |

Notes: Time-varying predictors were partitioned into three levels i.e., centered within context. These included: assessment-level effects (*level-1*; also within-day effects), which reflect deviations of an individual assessment’s raw score from the day-level mean; day-level effects (*level-2;* also between-day effects), which indicate deviations of the day-level mean from the person-specific mean; and person-level effects (*level-3;* also between-person effects), represented by person-specific means (i.e., each person’s average score across all assessments during the study period).

*** *p*<.001.


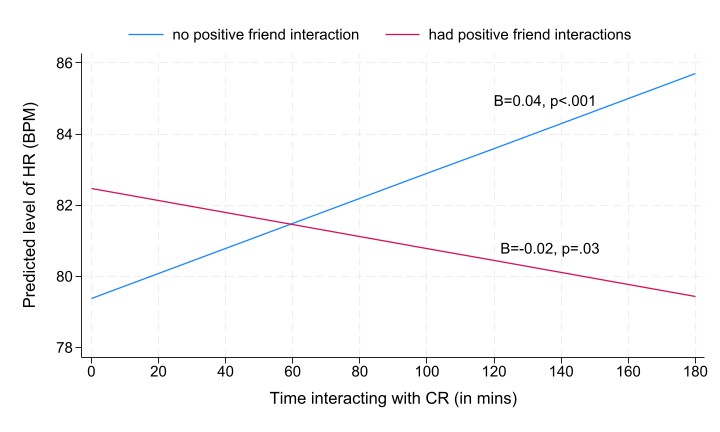
**Supplementary Figure 1.** **Within-day effects of CR time on heart rate in Demantia caregivers by any positive interaction with friends.** When caregivers did not have any positive interactions with friends (blue line), more CR interaction time was associated with increased HR. With positive friend interaction, more CR interaction time was linked to decreased HR (red line).


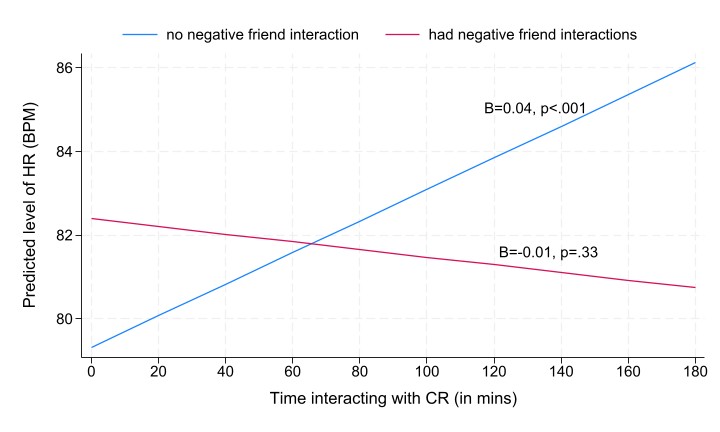
**Supplementary Figure 2.** **Within-day effects of CR time on heart rate in Demantia caregivers by any negative interaction with friends.** When caregivers did not have negative interaction with friends (blue line), more CR interaction time was associated with increased HR. With negative friend interaction, more CR interaction time was linked to decreased HR (red line).


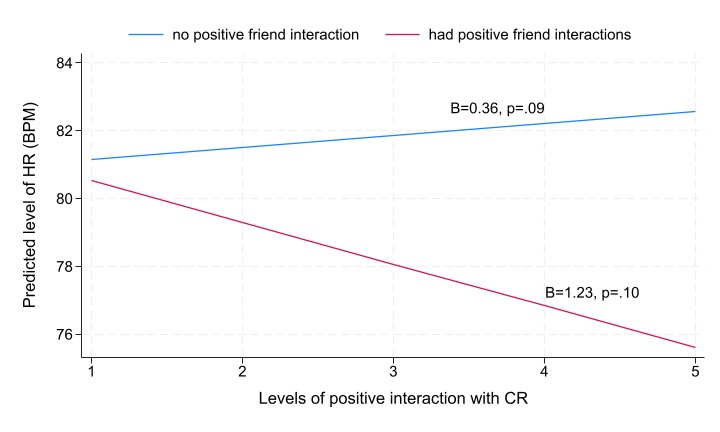
 **Supplementary Figure 3. Within-day effects of levels of positive interaction with CR on heart rate in Demantia caregivers by any positive interaction with friends.** Higher levels of positive interactions with CRs were showing a trend of reduced HR when caregivers also had positive interactions with friends (red line). When caregivers did not have any positive interaction with friends, higher levels of positive interactions with CRs were showing a trend of increased HR (blue line). Although these simple slopes were not significantly different from zero, their slope differences were significant.


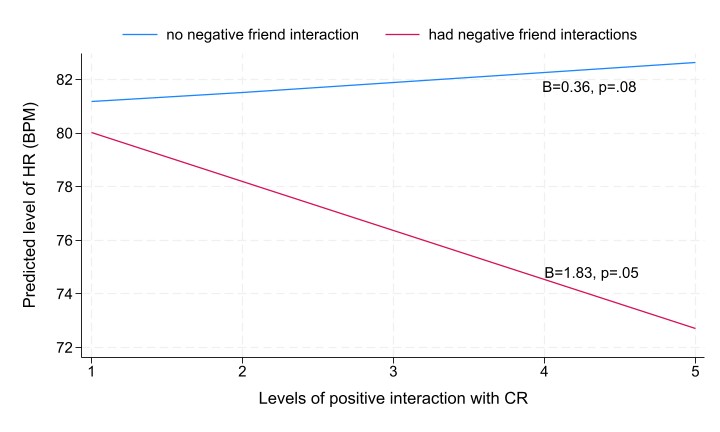


**Supplementary Figure 4. Within-day effects of levels of positive interaction with CR on heart rate in Demantia caregivers by any negative interaction with friends.** Higher levels of positive interactions with CRs were showing a trend of reduced HR when caregivers also had negative interactions with friends (red line). When caregivers did not have any negative interaction with friends, higher levels of positive interactions with CRs were not associated with increased HR (blue line). Although these simple slopes were not significantly different from zero, their slope differences were significant.
